# Moment-to-moment dynamics of ADHD behaviour in South African children

**DOI:** 10.1186/1744-9081-2-11

**Published:** 2006-03-28

**Authors:** Heidi Aase, Anneke Meyer, Terje Sagvolden

**Affiliations:** 1 Norwegian Institute of Public Health, Division of mental health, P.O Box 4404 Nydalen, N-0403 Oslo, Norway; 2 School of Health Sciences, University of Limpopo, South Africa; 3 Department of Physiology, University of Oslo, Norway; 4 Centre for Advanced Study at the Norwegian Academy for Science and Letters, Oslo, Norway

## Abstract

**Background:**

The behaviour of children with Attention-Deficit/Hyperactivity Disorder is characterized by low predictability of responding. Low behavioural predictability is one way of operationalizing intra-individual ADHD-related variability. ADHD-related variability may be caused by inefficient behavioural selection mechanisms linked to reinforcement and extinction, as suggested by the recently published dynamic developmental theory (DDT) of ADHD. DDT argues that ADHD is a basic neurobehavioural disorder, caused by dysfunctioning dopamine systems. For establishing ADHD as a neurobehavioural disorder, findings from studies conducted in Western countries should be replicated in other cultural populations. The present study replicated the study conducted in Norway, with children from the Limpopo province in the Republic of South Africa.

**Methods:**

Boys and girls, aged 6–9 yr, from seven ethnic groups participated. Scores by teachers on the Disruptive Behavior Disorders rating scale defined participation in either ADHD-hyperactive/impulsive (-HI), ADHD-predominantly inattentive (-PI), or ADHD-combined (-C) groups. Children below the 86^th ^percentile were matched on gender and age and comprised the non-ADHD group. The children completed a computerized game-like task where mouse clicks on one of two squares on the screen resulted in delivery of a reinforcer according to a variable interval schedule of reinforcement. Reinforcers were cartoon pictures presented on the screen together with a sound. Predictability of response location and timing were measured in terms of explained variance.

**Results:**

Overall, the results replicated findings from Norway. Specifically, the ADHD-C group showed significantly lower predictability of responding than the non-ADHD group, while the ADHD-HI and the ADHD-PI groups were in-between. In accordance with the previous study, response location, but not response timing, was a sensitive behavioural measure. There were no significant gender differences. Cartoon pictures were effective reinforcers as the non-ADHD group showed learning of the task. There was no relation between behavioural predictability and motor functions.

**Conclusion:**

The present study makes a strong case for ADHD as a basic, neurobehavioural disorder, not a cultural phenomenon, by replicating findings from a wealthy Western country in a poor province of a developing country. The results were, generally, in line with predictions from the dynamic developmental theory of ADHD by indicating that reinforcers were less efficient in the ADHD group than in the non-ADHD group. Finally, the results substantiated ADHD-related variability as an etiologically important characteristic of ADHD behaviour.

## Background

Attention-deficit/hyperactivity disorder (ADHD) [1] is a behavioural disorder affecting about 2–5% of grade school children [2], making it one of the most common child psychiatric diagnoses in the United States of America and in Europe. In childhood, the diagnosis is more frequent among boys. Depending on the referral practice, boys to girls ratios vary between 10:1 (clinical samples) and 3:1 (community samples) [3]. Research indicates that the major aetiological factor is genetic [4], probably mainly expressed as alterations in catecholaminergic regulation of brain activity [5,6]. The disorder places the child at risk for school failure and dropout, juvenile delinquency, criminality, substance abuse, and sexual promiscuity with HIV/AIDS and teenage pregnancies as possible consequences. In this way, the disorder is extremely costly, both to the afflicted individuals and their families, and to the society [7,8]. Although it has been discussed whether ADHD is a phenomenon of the Western culture, e.g., [9], its worldwide existence is well documented [10-13]. However, it seems that inconsistent assessment criteria and procedures affect prevalence rates of ADHD across countries and cultural groups [10]. The reliance on behavioural observations and clinical descriptions of the behaviour makes the diagnosis vulnerable to subjective and cultural perceptions, and the need for more objective criteria for diagnosing is long needed. No biological marker has yet been found, and no existing neuropsychological test can reliably define a case of ADHD [14]. However, theory-driven research aimed at identifying dysfunctions in basic behavioural mechanisms may provide an empirical basis for understanding processes and functions on other levels of analysis (e.g., developmental, neuropsychological, psychosocial), and for generating more sophisticated tests for early and reliable identification of affected individuals. The main purpose of the present study is to replicate an earlier study [15] showing that moment-to-moment dynamics of ADHD behaviour may represent a new way of understanding underlying behavioural mechanisms basic to ADHD.

ADHD is characterized by age-inappropriate hyperactivity, impulsiveness, and deficient sustained attention [1]. Most clinical and experimental reports show increased variability, both between and within subjects in the ADHD group. While the group heterogeneity suggests multiple independent pathways to the disorder [16], the intra-individual variability might be a key characteristic of an endophenotype of ADHD and has recently become a topic of particular interest [15-17]. A purportedly important step in the direction of describing and explaining the role and function of this variability is more fine-grained analyses of behaviour as opposed to the traditional statistical summary measures of means and standard deviations [18]. In the present study, response data is analyzed on a microlevel in order to identify possible mechanisms underlying the observed intra-individual variability.

ADHD-related variability is mainly observed at the behavioural level [16], so a thorough behavioural account is warranted in order to guide the investigation of, for instance, underlying neurobiological processes. Castellanos and colleagues [16] raised a number of key questions about ADHD-related variability that need to be addressed in a basic research programme, including its robustness in its association with ADHD; whether it is a random or dynamic-periodic phenomenon, whether it varies dynamically as a function of context, whether it is unique to ADHD, and finally, whether it reflects processes causal to ADHD. In the recently published dynamic developmental theory of ADHD (DDT) [19] it is argued that the main behavioural selection mechanisms, reinforcement and extinction, are altered in ADHD. These alterations bring about a different learning style resulting in increased behavioural variability, in addition to hyperactivity, impulsiveness, and deficient sustained attention. Some of the variability may be the result of deficient acquisition of reinforced behaviour combined with deficient extinction of non-reinforced behaviour causing shorter and less predictable behavioural sequences [15,19]. Thus, the DDT places ADHD-related variability within a causal model [19]. The DDT thereby suggests that ADHD-related variability is not a random phenomenon, but can be predicted in the combined and the hyperactive/impulsive subtypes of ADHD. Further, the DDT argues that ADHD-related variability will vary dynamically as a function of context, task, and motivational preferences. Recently, we showed that boys with ADHD combined and hyperactive/impulsive subtypes had significantly less predictable response sequences than normal boys [15] and that the overall variability was observed during infrequent reinforcement as opposed to frequent [20]. However, only boys from a culturally homogeneous population participated, so it is of vital importance that the phenomenon can be replicated in other samples.

The present study is a replication and extension of that study, with a larger sample including both genders. Further, the study is conducted in a developing country with a culturally less homogeneous population. However, clinical resources are sparse, and assessment methods developed and validated in Western countries may not be relevant or feasible. A recent prevalence study conducted in various language groups in South Africa showed that when using a teacher rating scale, similar figures for prevalence as in Western countries were obtained. Also, rather similar distributions across the three ADHD subtypes (inattentive, hyperactive/impulsive, and combined) were found, in addition to a similar gender distribution [12].

The primary aim of the present study was to investigate if the results from the Norwegian study could be replicated in a different sample derived from various language groups in the Limpopo province of South Africa. If replicated, the combined results from the Norwegian and the South African studies would make a strong case for a basic, neurobiological mechanism differentiating ADHD from non-ADHD children, refuting the argument that ADHD is a cultural phenomenon resulting from Western way of life, or from Western conceptualization of psychiatric problems. In addition, the findings might lend support to the recently published dynamic developmental theory of ADHD [19] arguing that the main behavioural selection mechanisms, reinforcement and extinction, are altered in ADHD and will result in increased behavioural variability.

The present sample made it possible to investigate potential differential effects of reinforcers on response patterns in the three subtypes of ADHD [2]: the predominantly inattentive type (ADHD-PI), the hyperactive/impulsive type (ADHD-HI), and the combined type (ADHD-C). In addition, potential gender differences could be studied.

ADHD-related variability was defined as reduced predictability of consecutive responses. The task used was a computerized game where mouse clicks on either of two squares on the screen resulted in the presentation of a reinforcer. Reinforcers were cartoon pictures displayed on the screen for a short period accompanied by a sound. These were delivered according to variable interval (VI) schedules of reinforcement. VI schedules specify that responses result in a reinforcer after varying time intervals. Thus, reinforcers are presented at unpredictable times [21], avoiding any confounding with time discrimination problems. All mouse clicks were recorded both in terms of where on the screen responses were placed (response location; the spatial dimension) and response timing (the temporal dimension). Thus, the present task allowed for analysis of both spatial and temporal aspects of behaviour.

## Methods

### Participants

Children from seven ethnic groups of the Limpopo Province of South Africa (Northern Sotho, Venda, Tsonga, Tswana, North Ndebele, Afrikaans, and English) were recruited from a school-based population. The 179 children (128 boys and 51 girls) were recruited following screening of the general population of primary school children representative of all socio-economic levels for ADHD-like behaviour. The Disruptive Behavior Disorders (DBD) rating scale [22,23] was standardized for the populations of the Limpopo province of South Africa in an earlier study [12] and used as the screening instrument. Both teachers and parents were given the rating scale to complete. Only the teacher's ratings were used for the screening, since the return of the parent's rating scale was below 50%, probably because many children either did not live with their parents or the parents were illiterate. Teacher ratings are usually regarded as an accurate measure of assessment [24,25]. The teachers returned ratings of close to 100% of their pupils. The children meeting the criteria for inclusion into the clinical group (~7%) were selected for further testing. They were matched for gender, age, and language with children without ADHD as measured by the screening process.

Children were divided into a group classified as ADHD, the "ADHD group" and a comparison group without ADHD symptoms (Table 1), based on teacher ratings on the DBD rating scale. The cut off point for the *ADHD *group (95^th ^percentile or above) was based on the results from the prevalence study [12] in which more than 6000 children in the Limpopo Province were rated on the DBD scale. Norms were developed for a high cut-off based on the qualified assumption that ADHD exists in about 5% of the population [12]. According to these norms, scores higher than 18 on the hyperactive/impulsive (H/I) items were classified as *ADHD-HI *and higher than 21 on the inattentive (Inatt) items were classified as the *ADHD-PI *group. If the criteria were met on both types of items, the child was classified as *ADHD-C*. The cut off point for the *comparison *group was set at the 85^th ^percentile or below in order to decrease the risk for false negatives in this group, as the DBD rating was the only measure. Thus, children with scores on H/I-related items less than 15 *and *Inatt items less than 17 were regarded as comparisons.

**Table 1:** Means and Standard Deviations, for Age and DBD Scores, by Subtype

	***ADHD-C***		***ADHD-HI***		***ADHD-PI***		***Non-ADHD***		
	*Boys*	*Girls*	*Boys*	*Girls*	*Boys*	*Girls*	*Boys*	*Girls*	**Total**
Age (mo)*	102.4 ± 10.6	104.4 ± 11.8	109.5 ± 20.7	103.6 ± 9.0	104.5 ± 15.7	102.9 ± 19.2	108.0 ± 14.9	101.5 ± 11.8	106. 4 ± 5.1

DBD** Inatt	24.0 ± 3.8	23.8 ± 2.7	13.8 ± 4.2	14.8 ± 2.5	22.2 ± 4.4	25.8 ± 2.6	6.6 ± 5.7	5.2 ± 5.9	13.5 ± 9.2

DBD** H/I	22.0 ± 3.2	20.9 ± 3.2	20.2 ± 1.8	20.6 ± 2.1	10.0 ± 6.0	9.9 ± 4.1	4.1 ± 3.6	4.4 ± 5.1	10.6 ± 8.4

***N***									
Afrikaans	2	3	1	1	3	1	10	5	26
English	0	0	0	0	1	0	2	1	4
N Sotho	1	3	4	1	0	1	12	9	31
Tsonga	3	1	3	1	8	2	3	1	22
Venda	4	2	2	4	11	2	17	8	50
N Ndebele	3	4	3	0	4	2	8	3	27
Tswana	3	0	3	1	1	0	9	2	19

**Total**	16	13	16	8	28	8	61	29	179

* There were no statistically significant differences in age between the subtypes

** Differences between groups were not tested, as the groups were defined by these scores.

DBD: Disruptive Behavior Disorder rating scale [22]; Inatt: Scores on inattentive items; H/I: Scores on hyperactive/impulsive items

The final sample consisted of children from seven ethnic groups inhabiting the Limpopo Province of South Africa. For the Afrikaans and English speaking groups, the IQ was established with the Senior South African Individual Scale (SSAIS-R) [26]. As there are no standardized IQ tests for the indigenous African populations, Raven's progressive matrices were used to estimate IQ. This test is also considered to be "culture-fair" [27,28]. Children with IQ lower than 80 and/or with a history of neurological problems (e.g. epilepsy, head injuries, cerebral palsy, or cerebral malaria) were excluded. None of the children were on psychostimulant medication at the time of testing.

### Procedure

Written permission was obtained from the Department of Education, Limpopo Province, as well of the principals of the selected schools. Participation was voluntary. Informed consent was obtained from the child's parents or guardians.

The children were always tested by a tester fluent in the child's own language. Most assessments were done at their schools during school hours, where one of the classrooms was made available. The exceptions were children whose school was within a radius of 2 km from the University and children referred for clinical assessment. These were tested at the University Clinic.

The SSAIS-R IQ test [26] and the Raven Progressive Matrices Test were administered by Masters students in Clinical Psychology who were doing their hospital internship.

As many of the children were not acquainted with computers, a 'mouse-training' session was part of the testing procedure.

### Reinforcement task

The task was designed as a computer "game" and was run on one of three similar laptops. The screen resolution was set to 640 by 480 pixels. The response device was a standard computer mouse and clicks on either left or right button were recorded as responses. Moving the mouse made the cursor move on the screen, the cursor being in the shape of an arrow. Two squares (140 × 140 pixels) 120 pixels apart, one in a light and one in a dark shade of grey, were displayed on the screen 120 pixels from the vertical sides (left-right) and 170 pixels from the top and from the bottom. A click within one of the squares induced a brief change in colour as feedback; the dark grey square turned into a lighter shade and the light grey square turned into a darker shade. Clicks outside the squares were recorded, but gave no feedback. Following reinforcer presentations, the two squares switched sides at random, but were never displayed on the same side more than twice in a row. The number of presentations on each side was the same.

The task was designed according to a multiple variable interval (VI) schedule of reinforcement. A schedule is called multiple when two or more schedule components alternate and are signalled by discriminative stimuli. In an interval schedule some time must elapse before a response will result in delivery of a reinforcer. In VI schedules, the intervals will vary around a specified arithmetic mean [21]. The reinforcer-dense schedule was a VI 2 s and the reinforcer-lean schedule was a VI 20 s schedule of reinforcement. The screen's background colour changed according to the contingency in operation and functioned as the conditioned discriminative stimulus for the two contingencies. A navy blue background signalled the VI 2 s, while a yellow background signalled the VI 20 s. The dark grey square was always associated with reinforcement (the correct square), thus this was the discriminative stimulus. Reinforcers were cartoon pictures displayed on the screen for 1.5 s simultaneously with a sound (different computer-generated sounds).

The child was introduced to the test with the following instruction (told in the child's own native language):

"This is a game you may play now. It is a little strange, because I will not tell you how to play the game. Your task is to find out how the game works. You may use this mouse and move the arrow across the screen like this (experimenter demonstrates how to move the mouse and cursor). If you want to point, you can click with one of these buttons (experimenter points to the mouse buttons). You may talk while you are playing, but I will not answer any questions about the game. I will sit back here and write a little while you play. Do you understand your task? You may start now."

The task started with a shaping sequence where every correct response was reinforced. The screen background was blue and the correct square was always on the same side (right). After six correct responses, the VI 2 s schedule came into effect without any signal. The child received four reinforcers upon responding during the VI 2 s contingency before the schedule changed to the VI 20 s contingency (and the background changed from blue to yellow) where four reinforcers were to be obtained. The time it took to obtain these eight reinforcers constitute the first segment of the test. The schedules alternated so that each was displayed five times; i.e., there were five segments, resulting in a total of 40 reinforcers per child (plus six from the shaping sequence). The entire task, including instruction and shaping, took about 10–13 min to complete.

### Data recording and statistics

Data were recorded by the laptops. Percentage of all responses within the correct square, response side (left or right), response coordinates (i.e. the horizontal and vertical pixel that the tip of the arrow-shaped cursor touched when the child clicked a mouse button), and interresponse times (IRTs) were the recorded dependent variables. The individual IRT distributions were highly skewed with a long tail towards long IRTs. IRTs were therefore normalized by log transformations prior to analysis [logIRT = log10 ((IRT/1000) + 0.001)].

#### Behavioural measures

The same behaviours were analyzed in the previous [15] and the present study. Response sequences were analyzed in the VI 20 s condition only; as the short schedule usually would not allow for enough responses in a segment (i.e., four reinforcer deliveries within the VI 2 s condition) to calculate autocorrelations and explained variance (see below). The following behaviours were analyzed: 1) A general *side response pattern*, i.e., whether consecutive responses were on the left or right side of the screen. Highly predictable responding would typically be on the side where the correct target was positioned, and indicates good stimulus control. Likewise, low predictability implies that responses are unsystematically distributed on the two sides and means poor stimulus control. 2) The *distance-to-nearest-centre *measure was based on the distance from the pixel where the response was placed, to the centre of the selected square, whether it was the correct square or not. This measure indicates whether the children developed strategies of responding that was related to the borders of the squares, disregarding whether the square was correct or not. 3) The *distance-to-correct-centre *measure was based on the distance from where the response was placed to the centre of the correct square. Thus, this measure was a variant of the distance-to-nearest-centre measure, anchored to the centre of the correct square. Both distance scores (measure 2 and 3) were in terms of vertical and horizontal pixels, with 0,0 defining the centre of the square. 4) *Timing response patterns *were based on consecutive interresponse times (IRTs).

The predictability of responses spatially and temporally was assessed by explained variance, i.e., autocorrelations squared. Explained variance is a better measure for overall predictability than autocorrelations by itself since the latter will have both positive and negative values cancelling each other when added. Autocorrelations (serial correlations) of each measure were correlations of consecutive response measures across five lags. Thus, correlations between the value of response n and of response n+1 is the first lag, between n and n+2 is the second lag, and so on up to correlations between response n and n+5 being the fifth lag. For programming reasons, autocorrelations were computed on consecutive responses through a full segment and not reset at reinforcer delivery because the number of responses was huge compared to the number of reinforcers. Changes in predictability of responding throughout the experiment could be observed in the explained variance for each child from segment to segment. An increase in autocorrelations over segments would indicate that performance became more and more predictable throughout the experiment, and thus be an indirect measure of learning. We hypothesised that compared to the non-ADHD group less of the behaviour of children with ADHD would be predictable, indicated by generally less explained variance. In addition, predictability of responses in the ADHD group should, to a larger extent than in the non-ADHD group, be restricted to the first lag, indicating shorter behavioural sequences.

In addition to the response sequences, *learning *was measured as mean percent of all responses that were placed within the correct square. A high score on percent correct would indicate that the dark grey square exerted good stimulus control over the responding of the children, and is thus a measure of attention.

#### Statistics

Data were analyzed by means of SPSS 11.0 for Windows (SPSS) and Statistica 6.1 [29] program packages. The distance scores were computed as the square root of the sum of squared horizontal and vertical distances. Explained variance (autocorrelations squared) was analyzed using repeated measures ANOVA across segments and lags. The ANOVA was supplemented with MANOVA. A multivariate approach to repeated measures is recommended when variables have more than two levels because MANOVAs correct for the assumption of compound symmetry and sphericity in ANOVAs [29].

Analyses relevant for the primary aim were performed first, with Group (2: ADHD and non-ADHD) as the between-group variable; and segment (5) and lag (5) as within-group variables. The ADHD group consisted of children with ADHD-C and ADHD-HI in order to replicate the Norwegian study. Then, as follow-up analyses, subtypes of ADHD (ADHD-HI, ADHD-PI, and ADHD-C) versus non-ADHD were analysed across segments and lags, in order to investigate potential differences between subtypes of ADHD. Results were followed up with post hoc Scheffé tests where relevant. Non-published results may be obtained from the first author upon request.

#### Demographic data

Demographic and diagnostic measures of the ADHD-related subtypes and the non-ADHD comparison group are displayed in Table 1.

## Results

Generally, there was no effect of Gender, neither main effects nor interaction effects. Consequently, the reported findings combine boys and girls. Further, predictable responding was found for the three spatial behavioural measures, but not for the timing measure. The ADHD Combined group had the lowest explained variance of all groups on the spatial measures.

### Side response pattern

This measure assessed whether the child's choice of side of the screen (left or right) was predictable irrespective of on which side the correct response target was displayed. Highly predictable responding across lags would imply that behaviour was orderly related to side of screen.

**Figure 1:**
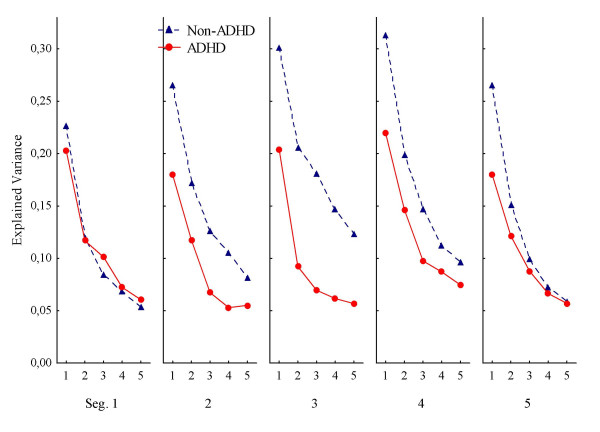
**Response predictability according to side of the screen**. Predictability of consecutive responses according to side of the screen (left or right), depicted as mean explained variance (autocorrelations squared), across segments (Seg. 1–5) and lags (1–5 per segment), for ADHD and non-ADHD groups. Abbreviations: Seg.: segment of session. Lag: number of responses that has to be correlated to the present one, i.e., correlations between response n and n+1 is the first lag, between n and n+2 is the second lag, and so on up to correlations between response n and n+5 being the fifth lag.

Generally, predictability from one response to the immediate next (first lag) was in the low range (0.31 > mean R^2 ^> 0.17; median R^2 ^= 0.22) compared to previously published results [15]. The side response pattern of the non-ADHD group was more predictable compared to the ADHD group (Fig. 1). There were significant main effects of Group and of Segment (Table 2). The ANOVA showed significant interaction effects between Group and Segment, between Group and Lag, and between Group, Segment, and Lag. All interactions were confirmed with the multivariate analysis (Table 2). The significant main effect of Segment implies that there was a general upward trend in predictability from segment 1 to segment 4.

**Table 2:** Results from repeated measures ANOVA and multivariate tests for repeated measures, of explained variance (squared autocorrelations)

***Measure***	***Variable***	ANOVA		Multivariate	
		
		Df	F	Df	F
**Side Response Pattern**	Group (G)	1, 146	13.857*		
	Segment (Seg)	4, 584	3.769**	4, 143	2.650*
	Lag	4, 584	167.626***	4, 143	49.352***
	G * Seg	4, 584	3.941**	4, 143	2.792*
	G * Lag	4, 584	4.206**	4, 143	2.697*
	G * Seg * Lag	16, 2336	1.802*	16, 131	2.090**
					
**Distance to centre of nearest square**	G	1, 146	2.588		
	Seg	4, 584	0.150	4, 143	0.146
	Lag	4, 584	135.577***	4, 143	41.892***
	G * Seg	4, 584	0.425	4, 143	0.428
	G * Lag	4, 584	1.242	4, 143	2.863*
	G * Seg * Lag	16, 2336	1.410	16, 131	1.487
					
**Distance to centre of correct square**	G	1, 146	3.974*		
	Seg	4, 584	0.875	4, 143	0.723
	Lag	4, 584	187.974***	4, 143	57.819***
	G * Seg	4, 584	4.475***	4, 143	3.663**
	G * Lag	4, 584	4.996***	4, 143	1.943
	G * Seg * Lag	16, 2336	1.505	16, 131	1.203
					
**Timing Response Pattern**	G	1, 146	0.843		
	Seg	4, 584	1.270	4, 143	0.929
	Lag	4, 584	60.096***	4, 143	31.501***
	G * Seg	4, 584	0.994	4, 143	0.944
	G * Lag	4, 584	0.660	4, 143	0.922
	G * Seg * Lag	16, 2336	1.107	16, 131	1.374

*: p < .05; **: p < .01; ***: p < .001

The follow-up ANOVA of the three ADHD subtypes and the non-ADHD group showed no significant main effect of Subtype, but the main effects of Segment and of Lag were significant. There was a significant interaction between Subtype and Lag (F(12, 720) = 2.99; p < .001), and between Subtype, Segment, and Lag (F(48, 2880) = 1.62; p < .005). Only the three-way interaction was confirmed by the MANOVA. The interaction effects implied that predictability in responding for the three ADHD subtypes and the non-ADHD group changed differently across segments and lags. The ADHD-C group showed the least predictable responding across segments. No other interaction effects were statistically significant.

### Distance to nearest centre

This measure assessed to what degree the children responded in predictable patterns in terms of the distance from where the response was placed to the centre of the nearest square irrespective of whether it was the correct response target or not.

**Figure 2:**
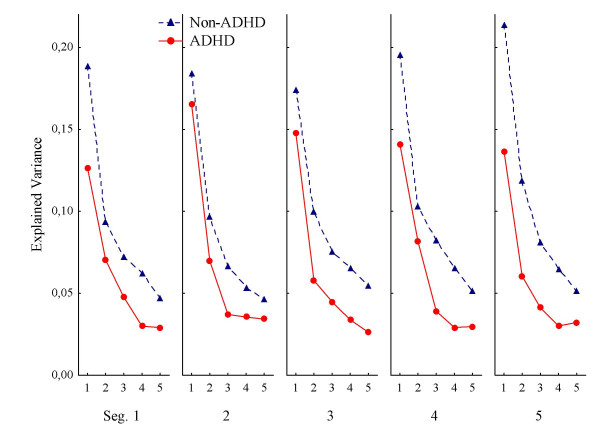
**Response predictability according to distance from the centre of a square**. Predictability of consecutive responses according to distance from the centre of a response square, whether correct or not, to where on the screen the responses were placed. Curves show mean explained variance (autocorrelations squared) across segments (Seg. 1–5) and lags (1–5 per segment), for ADHD and non-ADHD groups. Abbreviations: Seg.: segment of session. Lag: number of responses that has to be correlated to the present one, i.e., correlations between response n and n+1 is the first lag, between n and n+2 is the second lag, and so on up to correlations between response n and n+5 being the fifth lag.

The generally low values of explained variance in the first lag (0.21 > mean R^2 ^> 0.12; median R^2 ^= 0.17) show a rather low predictability from one response to the immediate next. As can be seen in Figure 2, the ADHD group was generally lower than the non-ADHD group. There was no significant main effect of Group, however, but a significant main effect of Lag. The interaction between Group and Lag was significant in the multivariate analysis (Table 2).

The follow-up ANOVA of the three subtypes and the non-ADHD group showed no significant effects except a significant main effect of Lag (F(4, 720) = 149.27; p < .001). In order to check if there were statistically significant differences between any two subtypes, a post hoc Scheffé test of Subtype and Lag was performed. The main results from this test indicate a larger decrease in explained variance from the first to the next lags in the non-ADHD group and in the ADHD-PI subtype compared to the other subtypes, and that there was a larger difference between the ADHD-C subtype and the non-ADHD group compared to any other combination of subtypes.

### Distance to correct centre

**Figure 3:**
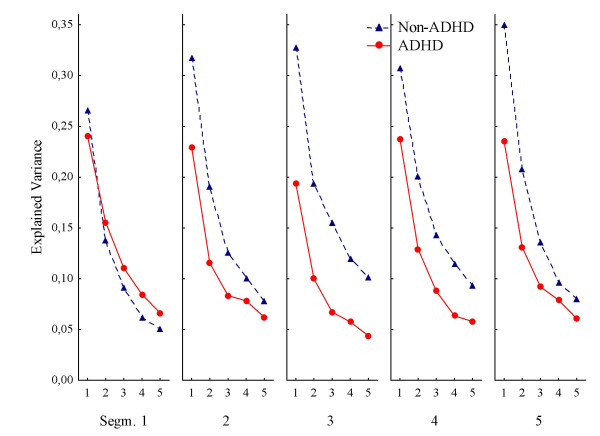
**Response predictability according to distance from the centre of the correct target**. Predictability of consecutive responses according to distance from the centre of the correct target square to where on the screen the responses were placed. Curves show mean explained variance (autocorrelations squared) across segments (Seg. 1–5) and lags (1–5 per segment), for ADHD and non-ADHD groups. Abbreviations: Seg.: segment of session. Lag: number of responses that has to be correlated to the present one, i.e., correlations between response n and n+1 is the first lag, between n and n+2 is the second lag, and so on up to correlations between response n and n+5 being the fifth lag.

This measure assessed patterns in response placements in terms of distance from the centre of the correct square. Again, low explained variance indicated high variability in spatial responding. As can be seen in Figure 3, responding was somewhat more predictable with this measure of behaviour compared to the two other spatial measures. Predictability from one response to the immediate next (explained variance in the first lag) was generally in the lower middle range (0.35 > mean R^2 ^> 0.19; median R^2 ^= 0.25) compared to previous results [15]. The ADHD group had less predictable behaviour than the non-ADHD group.

The ANOVA showed a significant main effect of Group, in addition to the main effect of Lag (Table 2). There were also significant interaction effects between Group and Segment, and between Group and Lag. The interaction between Group, Segment, and Lag did not meet conventional levels of significance (p > .08). Only the interaction involving Group and Segment was confirmed by the multivariate analysis (Table 2). This interaction showed that while the non-ADHD group's behaviour increased in predictability over segments, the ADHD group's behaviour did not improve over segments.

The follow-up ANOVA of the three subtypes and the non-ADHD group only showed a significant interaction effect between Subtype and Lag (F(12, 720) = 2.64; p < .002) in addition to a main effect of Lag (F(4, 720) = 198.39; p < .001). The interaction effect indicated that the ADHD-C group showed less behavioural predictability than any of the other groups, while the ADHD-PI did not differ from the non-ADHD group. The interaction was not confirmed by the MANOVA.

In order to check what contributed to the interaction effect, a post hoc Scheffé test was performed involving Subtype and Lag. The main results from this test confirmed some overlap between the non-ADHD and ADHD-PI subtypes, and that explained variance for these groups was significantly higher than that of the ADHD-C subtype.

### Timing response pattern

The development of patterns in response timing was investigated in terms of consecutive interresponse times (IRTs). Explained variance of the first lag was generally very low and not significantly different between the groups (0.08 > mean R^2 ^> 0.05). Besides a significant main effect of Lag, there were no other main or interaction effects (Table 2). The follow-up analysis of the subtypes showed a significant interaction effect between Group, Segment, and Lag (F(48, 2880) = 1.39; p < .05), which was not confirmed by the multivariate analysis. The interaction implied that patterns of explained variance changed between the groups both across segments and lags in an uninterpretable way. No further follow-up analyses were run for this measure.

### Learning

Percentage of all responses that were placed within the square associated with reinforcement (correct square) was used as a measure of learning. Overall, the non-ADHD group had higher percent correct scores than the ADHD group (58.6% vs 46.2%). The non-ADHD group increased their scores with about 10% (from 52.8% to 62.5%) across segments, while the ADHD group only increased with less than 2%. The ANOVA of percent correct choice of square showed significant main effects of Group (F(1, 147) = 16.142; p < .001) and of Segment (F(4, 588) = 7.635; p < .001). The interaction effect between Group and Segment was also significant (F(4, 588) = 4.297; p < .002); and was confirmed by the MANOVA (F(4, 144) = 2.964; p < .03).

**Figure 4:**
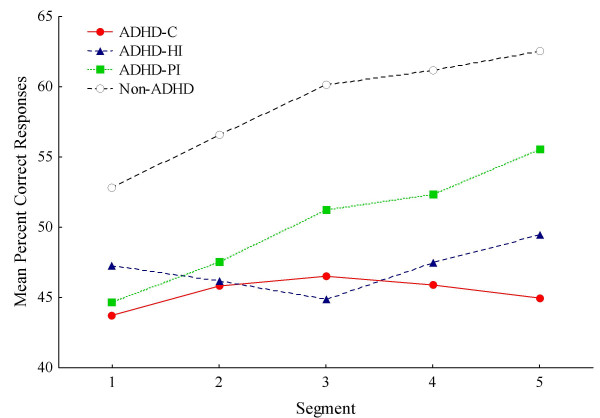
**Mean percent correct**. Mean percent correct choice of response target across consecutive segments (Seg) for ADHD and non-ADHD groups. Abbreviations: ADHD-C: ADHD combined type; ADHD-HI: ADHD hyperactive/impulsive type; ADHD-PI: ADHD predominantly inattentive type.

The follow-up ANOVA of the three subtypes and the non-ADHD group showed a main effect of Subtype (F(3, 180) = 6.14; p < .001) and of Segment (F(4, 720) = 7.18; p < .001) (Fig. 4). The interaction effect between Subtype and Segment did not meet conventional levels of significance (F(12, 720) = 1.72; p = .058). The main effect of Segment was confirmed by the MANOVA.

A post-hoc Scheffé test of the four subtypes showed that the non-ADHD group had significantly higher mean percent correct than the ADHD-C and the ADHD-HI subtypes (p < .008 and p < .04, respectively). The ADHD-PI group was not significantly different from any of the other subtypes, and the -C and -HI subtypes were not significantly different from each other.

### Response predictability and motor functions

A recent study of motor functions of the present South-African sample showed significant differences between children with ADHD-related symptoms and those without (Meyer & Sagvolden, subm. to BBF). Increased behavioural variability may be a result of motor coordination problems. This relation was tested by correlating first lag explained variance of all spatial measures with normalized scores on Grooved Pegboard test and Mazes (see Meyer & Sagvolden for details) for both dominant and non-dominant hand, for the ADHD-C group and the non-ADHD group separately. For the ADHD group, scores on response predictability was mainly negatively related to scores on motor tests (on the motor tests, low scores were preferred), but the relation was weak (Pearson correlations .234 > r > -.352). For the non-ADHD group, the relation was even weaker (Pearson correlations .237 > r > -.175).

## Discussion

The present study was designed to follow up the results of a study in a wealthy European country, Norway, which showed that response sequences in children with ADHD symptoms is less predictable than those of children without ADHD [15]. The present study was conducted in the poor Limpopo province in South Africa, a developing country with a more heterogeneous population, and with fewer resources and assessment options compared to Western countries. The children's behaviour problems were rated with the DBD [22] as psychiatric services are generally not available in developing countries. A strong case for ADHD as a basic, neurobehavioural disorder, not a cultural phenomenon, could be made if the results from Norway were replicated in a developing country.

The most striking finding in the present study was that the lower predictability of consecutive responses of boys with ADHD compared to controls in the Norwegian study [15] was replicated in boys and girls from the Limpopo province. Actually, when comparing the results from the two studies, the ADHD groups from the two populations seem more similar to each other than the non-ADHD groups are to each other. Combined, the results suggest that the phenomenon might pertain to the ADHD-C subtype in particular, although some results indicate that children with less severe ADHD, as those with hyperactive/impulsive subtype, may show weaker forms of the phenomenon. This conclusion is in line with predictions made from the dynamic developmental theory, DDT [19], applying specifically to ADHD-C and -HI subtypes.

Another striking similarity between the two studies was the finding that predictability of consecutive responding were found for the spatial behavioural measures only and not for the temporal measure. We speculated that this finding might be related to the visuo-spatial nature of the task, alternatively that striatal dysfunction might explain deficient habit learning in ADHD [15]. The fact that similar patterns of results were found in two extremely diverse samples, recruited by different methods and in different cultures, indicates a biologically-founded mechanism rather than a culturally-imposed response style, and that the mechanism might be specific to ADHD with symptoms of hyperactivity/impulsiveness. Imaging and neuropsychological research suggest a right hemisphere frontal-striatal circuitry involvement in ADHD, e.g., [30,31], indicating a specific dysfunction in perception and treatment of visuo-spatial stimuli. An alternative hypothesis, highly compatible with the DDT, has suggested that cortical basal-ganglionic neuronal modules learn to recognize and register complex contextual patterns that are relevant to behaviour, and that this learning is mediated by dopaminergic reinforcement signals [32]. The contextual information includes the state of the organism, the location of targets of action, the desirability of an action, motor intentions, and sensory inputs apt for either selecting or triggering motor programs. The pattern recognition going on in the striatum is guided by dopaminergic reward prediction signals [33]. The recursive process in the cortico-striatal module enables the basal ganglia to encode even more complex contexts based on those initially recognized [32]. With dysfunctioning dopamine systems, learning the association between different contextual cues and between environmental signals and relevant motor programs will be hampered [19].

It is important to notice that the cartoons acted as reinforcers in the non-ADHD children as shown by their learning curves (Fig. 4). Learning could also be observed on the distance-to-correct-centre measure (Fig. 3), showing increasingly better prediction from one response to the next (first lag) in the non-ADHD group over segments, but not in the ADHD group. The higher and increasing percent correct scores of the non-ADHD group, and the lower, flatter learning curves of the ADHD groups, replicate other findings from Norway [20]. In the present study the ADHD-C and -HI groups had more incorrect responses than correct throughout the session (Fig. 4). This indicates no stimulus control over behaviour by the dark grey square in these groups. Stimulus control is demonstrated when performance is predictably related to the stimulus signalling reinforcement, and the stimulus has gained conditioned reinforcing properties. For a stimulus to become a conditional reinforcer, it must be coupled with a primary reinforcer within a certain time, delimited by the length of a delay-of-reinforcement gradient (Catania's precommentary in [19]). The present findings may be explained by a short delay gradient in the -C and -HI groups, resulting in no association between the stimulus and the response-produced reinforcer, as predicted by the dynamic developmental theory [19]. For the ADHD-PI group, performance improved from about 45% to about 55% across segments and showed a parallel improvement to the non-ADHD group though at a 10% lower level overall. It might be that the non-ADHD group benefited more than the -PI group from the reappearing short VI segments, where reinforcers were presented more frequently, at approximately every 2 s (not shown), and thus learned the association with the dark grey square more quickly. Interestingly, the ADHD-PI group showed better attention than the other ADHD subtypes when using stimulus control as a measure of sustained attention (cf., [20]). This suggests that the clinically described attention problems characterizing the -PI subtype may be different from those often described in the -HI and -C subtypes [19,34]. The present findings thus support the assumption in the DDT that altered learning mechanisms related to a shorter delay gradient mainly apply to the -HI and -C subtypes and that the present attention measure mainly relates to the learning style of these subtypes in particular.

In general, the behaviour of all groups was less predictable in the present study than in the previous study [15], particularly when comparing the non-ADHD groups. Learning was also poorer, as mean percent correct never exceeded 63% even in the non-ADHD group, while the non-ADHD group in the Norwegian study performed up to 90% correct [20]. Several factors may explain this result. For instance, the groups were probably more heterogeneous in the present study. The DBD rating scale is most likely less sensitive than the standard comprehensive diagnosis performed in the Norwegian study. Also, the non-ADHD group was less well described and allowed for DBD scores up to the 85^th ^percentile. In addition, children in a developing country may not be as used to computers as Norwegian children. While most Norwegian children are acquainted with computer games, including clicking on items in order to bring up other items or happenings on the screen, the South African children may not be that familiar with computer games. This will result in more explorative and somewhat less systematic behaviour in the South African children. Further, an unintended procedural difference between the two studies might have influenced the results. In Norway, testers were instructed to add verbal feedback (like "Wow", "Look at that", etc.) when the cartoons were displayed on the screen. This was not done in South Africa. Thus the reinforcing effect might have been less, particularly for the non-ADHD children as this group deviated more from their Norwegian counterparts than the ADHD group.

The findings demonstrate, nonetheless, that the task could be run in just one session and with no extra tangible reinforcers. Hence, a quick (less than 15 min altogether), and easy task as the present actually showed basic behavioural differences between children with ADHD and children without symptoms.

The present study found few statistically significant differences between the three subtypes. When differences were indicated, they generally showed that the ADHD-C group performed with lower predictability than the other groups, and often the results for the ADHD-HI group fell between the -C and the -PI groups. The most likely explanation of this result is that it reflects that the behavioural disturbances in the ADHD-PI and ADHD-HI groups are less severe than those of the ADHD-C group.

There were no gender differences. This supports other findings with population-based samples, showing that non-referred subjects of both genders present with similar clinical and cognitive profiles [35]. These authors concluded that gender differences in comorbidities (including learning deficits) frequently found in clinical samples more likely are caused by referral biases and not by real differences between girls and boys with ADHD.

ADHD-related variability has been demonstrated in a plethora of tasks, particularly response time tasks [16]. These authors suggested that ADHD-related variability should be demonstrated simultaneously at different levels of analysis, like neurophysiological levels in addition to the behavioural; and, equally important, that such studies should be developed within a sound, theoretical framework. The present and earlier findings [15] suggest that predictability of response sequences is another potential operationalization of ADHD-related variability that might represent an etiologically important characteristic of ADHD. Further, these studies were designed within the framework of the dynamic developmental theory, a comprehensive theory of ADHD [19], arguing that the main behavioural selection mechanisms, reinforcement and extinction, are less efficient in ADHD. Specifically, the theory holds that altered reinforcement mechanisms; depicted by a shorter delay-of-reinforcement gradient, result in the accumulation of responses that are selected by both scheduled and unscheduled reinforcers. This occurs because consequences (which might be unscheduled or "accidental") in close proximity to a response will have a larger impact on future behaviour than a delayed consequence (which may be the planned or scheduled reinforcer). The finding that reinforcers affect the latest response more than an overall response pattern in ADHD (cf., [36]) supports this. Thus, immediate reinforcers may increase the future probability of any response that happened to be emitted before its delivery, resulting in augmented behavioural variability. In addition, dysfunctioning extinction mechanisms will curb pruning of inefficient (i.e., non-reinforced) responses so that behaviour that is no longer functional is retained in the person's behavioural repertoire for an extended period, thereby adding to the variability. The dynamic developmental theory relates the altered selection mechanisms of behaviour to dysfunctioning dopamine systems with corresponding predictions about other behavioural and neurobiological outcomes [19], which may prove valuable as correlational measures in future studies.

Increased variance may be a result of augmented motor difficulties in children with ADHD-related problems. In the Norwegian sample, scores on motor tests did not differentiate ADHD from non-ADHD groups [20]. Correlating first lag scores and scores on motor tests for the present ADHD group and the non-ADHD group did not show significant and systematic relations between motor functions and response predictability for any of the groups. Thus, deficient motor functions do not seem to be a significant predictor for predictability of response sequences in the task at hand.

The present task provides an objective measure of basic behavioural processes that is not confounded with timing skills, motor functions, performance requirements (including emotional reactions to failure experiences), or the correct understanding of more or less complicated instructions. As such, the task may prove easy to carry out; both for testers and subjects; and results are interpretable within a theoretical framework. Future studies will show its predictability and diagnostic utility.

Some obvious limitations to the present study should be mentioned. Group membership was decided solely on the basis of teacher scores on the Disruptive Behavior Disorders (DBD) rating scale [22,23] and not on a comprehensive diagnostic assessment. This might mean, for instance, that only the ADHD-C group, as defined by DBD-scores, captured "real" ADHD to a degree found through clinical evaluations. Inadequately defined groups most likely affected the results by bringing about increased within-group variability. Despite this, the present findings replicated to a large extent those from a well-described group from a different cultural background, and suggest that the task is sensitive to ADHD-related symptoms. However, the task has not been applied with other diagnostic groups than ADHD, so its specificity needs to be investigated. Finally, the findings only pertain to children within a narrow age range, which imply that future studies need to be conducted with more age groups.

## Conclusion

The present study makes a strong case for ADHD as a basic, neurobehavioural disorder, not a cultural phenomenon, by the overall replication of the results from Norway, a wealthy Western European country, in the very different, poor Limpopo province of South Africa and with a large majority of native African children.

Overall, the results were in line with the predictions from the dynamic developmental theory of ADHD by indicating that reinforcers were less efficient in the ADHD group than in the non-ADHD group in establishing stimulus control and predictable responding, due to a shorter delay-of-reinforcement gradient. The results also substantiated ADHD-related variability as an etiologically important characteristic of ADHD.

The present study did not find statistically significant differences between the non-ADHD group and the ADHD-HI or -PI groups. It is likely that this reflects less severe behavioural disturbances in these subtypes compared to the ADHD-C subtype.

In spite of basically similar response patterns, behaviour could not be predicted to the same degree in the South African children as in the Norwegian children, particularly when comparing non-ADHD groups. Likely reasons are less computer experience in South African children, procedural differences related to reinforcement, and increased group heterogeneity in the present study since the Disruptive Behavior Disorders (DBD) rating scale is less sensitive than the standard comprehensive diagnosis performed in the Norwegian study. Still, the DBD proves to be a powerful instrument.

Finally, there were no statistically significant gender differences. This supports other findings with population-based samples.

## Competing interests

The author(s) declare that they have no competing interests.

## Authors' contributions

HA participated in the development of study design, development of the reinforcement task used, participated in preparing the data, performed the statistical analyses, and wrote the manuscript. AM leaded the data collection, participated in preparing the data, participated in writing the manuscript, read and approved the final draft. TS participated in the development of study design, development of the reinforcement task, wrote the programs for statistical analyses, participated in data analyses, participated in writing the manuscript, read and approved the final draft.
